# Biomarker identification of isolated compartments of the cell wall, cytoplasm and vacuole from the internodal cell of characean *Nitellopsis obtusa*

**DOI:** 10.7717/peerj.10930

**Published:** 2021-02-17

**Authors:** Brigita Gylytė, Sigita Jurkonienė, Reda Cimmperman, Vaidevutis Šveikauskas, Levonas Manusadžianas

**Affiliations:** Institute of Botany, Nature Research Centre, Vilnius, Lithuania

**Keywords:** Biomarkers, Cell compartments, Cell wall, Cytoplasm, Vacuole, *Nitellopsis obtusa*, CuO nanoparticles

## Abstract

Cells of characean algae are attractive for plant cell physiologists because of their large size and their close relation to higher plant cells. The objective of our study was to evaluate the purity of the compartments (cell wall, cytoplasm with plastids, mitochondria, nuclei and endomembrane system, and vacuole) separated mechanically from the internodal cells of *Nitellopsis obtusa* using enzymatic markers. These included *α*-mannosidase and malate dehydrogenase, vacuolar and cytoplasmic enzymes, respectively. The biomarkers applied revealed the degree of compartment contamination with the material from unwanted cell parts. The cell wall was contaminated slightly by vacuole and cytoplasm residuals, respectively by 12.3 and 1.96% of corresponding biomarker activities. Relatively high activity of vacuolar marker in the cell wall could be associated with the cell vacuoles in the multicellular structure of the nodes. The biomarkers confirmed highly purified vacuolar (99.5%) and cytoplasmic (86.7%) compartments. Purity estimation of the cell fractions enabled reevaluating nCuO related Cu concentrations in the compartments of charophyte cell. The internalisation of CuO nanoparticles in *N. obtusa* cell occurred already after 0.5h. In general, the approach seems to be useful for assessing the accumulation and distribution of various xenobiotics and/or metabolites within plant cell. All this justifies *N.obtusa* internodal cells as a model organism for modern studies in cell biology and nanotoxicology.

## Introduction

Starry stonewort *Nitellopsis obtusa* is a benthic alga which is a bioindicator of clean fresh and brackish water bodies. The giant internodal stem cells of *N. obtusa* species belonging to the family Characeae are a suitable model for studying various responses to environmental factors including electrophysiological and enzymatic responses, and for cell survival experiments (Beilby & Shepherd, 1989; Beilby & Casanova, 2014; Gylytė et al., 2015). The internodal cell of *N. obtusa* has a cylindrical shape, typical to all characeans, and can grow to an approximate length of 30 cm and a diameter of 1–2 mm. Because of their large size and the close relation of Characeae to higher plants (Qiu, 2008; Nishiyama et al., 2018), internodal cells of *N. obtusa* are an excellent model for plant research (Turmel, Otis & Lemieux, 2003; Foissner & Wasteneys, 2014). Moreover, due to the huge vacuole that occupies 90–95% of the mature cell volume, rigid cell wall, and thin protoplasm layer it is possible mechanically separate these large subcellular compartments. Isolated compartments of characean cells have been used to study radioactively labelled metal accumulation (Hampson, 1967; Marčiulioniene et al., 2016), localisation and dynamics of metabolites in the vacuole (Oikawa et al., 2011) or identification of nanoparticles in specific cell compartments (Manusadžianas et al., 2017). Recently, internodal cells of *Chara australis*, along with other characeans, have been proposed as a model organism for modern biological studies at the single-cell level as well (Oikawa et al., 2011; Pertl-Obermeyer et al., 2018).

Single-cell fractionation is the process used to separate cellular compartments while preserving the individual functions of each component. Marker enzymes are used for determining the purity of cell fractions and the degree of contamination with unwanted parts of the cell: membranes, organelles or debris of nucleus, as well as to conclude whether isolated compartments remain functional following isolation procedures (Shimaoka, Ohnishi & Sazuka, 2004; Robert et al., 2007). Giant cells of Characeae have always been attractive to plant biologists. For example, based on an examination of vacuolar enzymes in *Chara corallina*, it has been suggested that the cytoplasmic proteases contribute to cellular protein turnover contrary to proteases in the central vacuole (Moriyasu, 1995). Plant vacuoles have various kinds of hydrolytic enzymes, including acid phosphatase, *α*-mannosidase, proteinase, carboxypeptidase and RNase (Kimura, Hess & Stur, 1999; Tan et al., 2019). *α*-Mannosidase activity has been used as a marker for intact vacuoles isolated from *Arabidopsis* suspension-cultured cells to validate the purity of the vacuole for each analysis (Ohnishi et al., 2018) as well as for vacuoles isolated from mesophyll leaf cells of tobacco and barley (Saunders & Gillespie, 1984; Kaiser et al., 1986). Chanda et al. (2009) have also confirmed *α*-mannosidase as a vacuole marker and showed a 35-fold enrichment of its activity in the vesicle-vacuole fraction of the filamentous fungus *Aspergillus parasiticus*. Thus, using this indicative vacuolar marker will allow quantitatively characterise possible contamination of other cell compartments by vacuolar sap during their isolation.

Generally, enzymes show compartment-specific localisation in plant cells. Dehydrogenases, including malate dehydrogenase, are one of the most extensively studied enzymes. NAD-dependent malate dehydrogenase is an enzyme very commonly occurring in plants (McMillin & Scandalios, 1980; Yudina, 2012). In particular, malate dehydrogenase activity is a cytoplasm marker, and its high activity makes the enzyme a reliable and convenient tool, which can effectively be used in the identification of this cell compartment (Chanda et al., 2009; Schreier et al., 2018). Besides, the purity of the isolated cytoplasmic solution has been assayed by malate dehydrogenase as a cytoplasm marker in macrophytic algae cells of *C. australis* (Oikawa et al., 2011). NADP-dependent isoforms of malate dehydrogenase were found in chloroplasts (Gietl, 1992; Selinski & Scheibe, 2019).

In our previous work, several fractions have been mechanically isolated from the cell of *N. obtusa*, namely cell wall, vacuole, the cytoplasm with chloroplasts and the mixture of vacuole and cytoplasm. Then cell fractionation technique has been applied to assess the location of accumulated CuO nanoparticles (Manusadžianas et al., 2017). However, to uphold the findings of this study, further specification of the procedure is needed, since the degree of possible contamination among the main cell compartments remained unclear. For example, although vacuolar sap had been verified by its higher acidity (pH5.4–5.5) (Gyenes et al., 1978) than that of cytoplasm, the presence of cytoplasm residues in the cell wall and/or vacuole compartments that could occur during the cell fractionation has not been taken into account. It is important to validate the cell fractionation procedure because charophyte cell is a suitable model for studying the localization of different toxicants and/or metabolites in the cell, and is of relevance for researchers working in plant cell physiology/toxicology (Schwab et al., 2015; Lv, Christie & Zhang, 2019). Therefore, the objective of our study was to re-examine the fractionation procedure, evaluate the purity of obtained fractions, and re-estimate the accumulation of the nCuO in the compartments of the internodal cells of *N. obtusa*. The enzymatic markers for fraction purity included *α*-mannosidase and malate dehydrogenase, vacuolar and cytoplasmic enzymes, respectively.

## Materials and Methods

### Algae material

**Figure 1:**
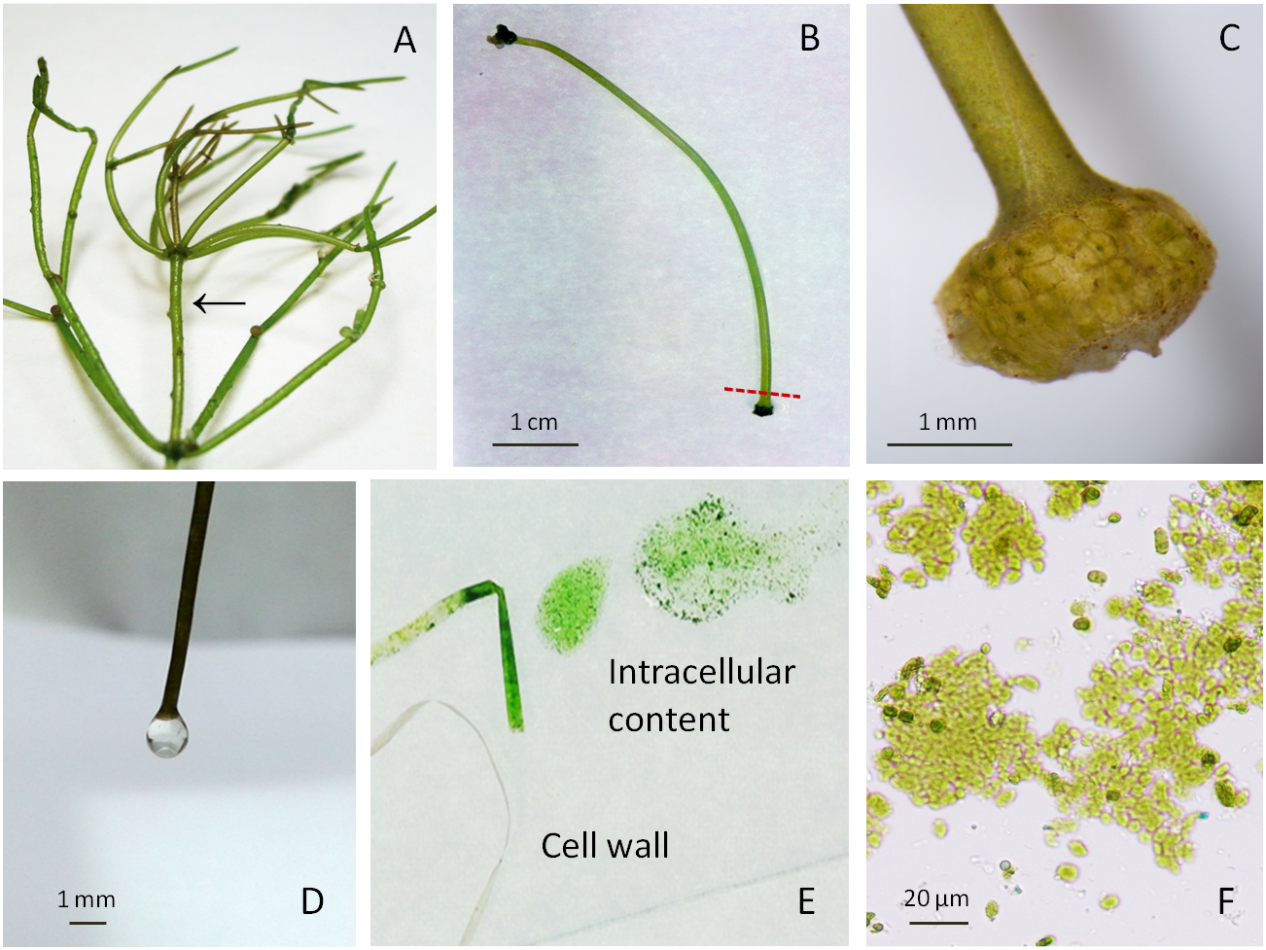
Macrophytic algae *Nitellopsis obtusa* and fractionation steps of the internodal cell. (A) Thallus; arrow shows a stem internodal cell. (B) Intact internodal cell; red dashed line shows an approximate place of dissection. (C) Internodal cell fragment with the node. (D) Drop of the vacuolar sap at the nodeless cell end. (E) Cell intracellular content extruded on the glass plate by squeezing and the cell wall. (F) Intact chloroplasts in a squeezed intracellular content.

Macrophytic algae *Nitellopsis obtusa* (Desv.) J. Groves, was collected from Lake Obelija (54°29′N, 23°83′E), south-east Lithuania. Separated from thallus (Fig. 1A), matured internodal cells, the 2nd, 3rd or 4th internode from the holdfast (each 10–25 cm in length), were stored at 18–24 °C in glass aquariums filled with equal parts of non-chlorinated tap water and artificial pond water containing (mM): 0.1 KH_2_PO_4_, 1.0 NaHCO_3_, 0.4 CaCl_2_, 0.1 Mg(NO_3_)_2_ and 0.1 MgSO_4_ (unbuffered, pH 7.0–7.8).

### Fractionation of internodal cells

The fractionation of internodal cell was mainly done as previously described in Manusadžianas et al. (2017). Specifically, a single cell was placed on a filter paper, air-dried for ∼1 min until the surface was opaque. One end of the internodal cell was cut with scalpel at approximately 4 mm distance from the node (Figs. 1B–1C), after the loss of turgor pressure, a condition in which a cell bends on the spatula and loses its cylindrical shape (Gylytė et al., 2015). One or two drops were obtained from the cell when it was cautiously held by the non-cut node in a vertical position above the microtube (Fig. 1D). The collected fraction of colourless sap represents the vacuole compartment. The internodal cells (15–20) were used to collect 0.51 ± 0.014 mL (mean ± sd, *n* = 3) of vacuolar sap. Then the rest of the cell intracellular content was squeezed by fingers along the cell surface up to the cut end (Fig. 1E). This fraction (0.96 ± 0.044 mL, *n* = 3) comprises the rest of the vacuole and cytoplasm with the organelles (Fig. 1F). After centrifugation at 10,000 rpm for 7 min (Beilby & Shepherd, 1989) (Labnet Prism™ R Refrigerated Microcentrifuge, Edison, NJ, USA), the obtained supernatant and residue (0.12 ± 0.04 mL, *n* = 3) were used for biomarker identification. Cell wall fraction consisted of cut nodes and what was left after the separation of intracellular content. Before enzyme activity measurements, the obtained cell walls were homogenised with deionised water (0.1 g tissue/0.4 mL water) and then immediately filtered through batiste material. Overall, a single internodal cell was separated into the following fractions: vacuolar sap, cytoplasm-vacuole mix (squeezed intracellular content without previously dripped vacuolar sap), the supernatant and the residue of the centrifuged cytoplasm-vacuolar mix, total intracellular content (all the squeezed cell interior content), and the cell wall. The pH of each fraction was measured with HI-1330 pH combination electrode (Hanna Instruments, Woonsocket, USA).

### Vacuolar marker assay

The purity of vacuolar fraction was examined measuring the activity of the specific marker enzyme *α*-mannosidase (Saunders & Gillespie, 1984; Kaiser et al., 1986; Chanda et al., 2009; Ohnishi et al., 2018). The activity of this enzyme was measured by a method adapted from Oikawa et al. (2011). 4-Nitrophenyl-a-D-mannopyranoside (PNP-a-Man) was used as a synthetic substrate for the *α*-mannosidase assay. Each of the fraction samples (30 µL with protein content 0.2–1.5 µg) was added to the reaction mix containing 50 µL of 5 mM PNP-a-Man and 100 mM citrate buffer (pH 5.6). After incubation at 37 °C for 1 h, the reaction was stopped by adding 750 µL of 200 mM Na_2_CO_3_ solution. The absorbance of the released p-nitrophenol was measured at 405 nm (Analytik Jena SPECORD®210PLUS, Jena, Germany). The specific activity was expressed in µmol of p-nitrophenol produced per min per mg of protein content.

### Cytoplasmic marker assay

Malate dehydrogenase activity was assayed as a marker enzyme for cytoplasm (Oikawa et al., 2011; Chanda et al., 2009). Malate dehydrogenase is an enzyme that catalyses the interconversion between malate and oxaloacetate by using NAD+/NADH as a cofactor. Malate dehydrogenase assay for each of the analysed fractions was performed using final concentrations of 50 mM HEPES (pH 7.5), 350 µM NADH, and 1 mM oxaloacetate in the reaction medium. The volume of 30 µL of the sample was added to 1 mL of reaction medium pre-incubated at 30 °C. Enzymatic activities were determined according to the NADH absorbance changes at 340 nm (Analytik Jena SPECORD®210PLUS, Jena, Germany) following the addition of the sample. Enzyme specific activity was expressed in pmol of malate formed per min per mg of protein content.

### Protein content measurement

Protein content was measured using the Bradford dye-binding procedure (Bradford, 1976).

### Photosynthetic pigments assay

The analysis of the content of photosynthetic pigments (chlorophyll a and b, and total carotenoids) in vacuolar and centrifuged vacuolar fractions of *N. obtusa* cells was performed after Wellburn (1994). Vacuolar and centrifuged vacuolar fractions were extracted with N, N-dimethylformamide (Sigma-Aldrich, Germany) at 4 °C in the dark for 24 h. The optical density of the extracts was measured at 480 nm, 647 nm, and 664 nm.

### CuO nanoparticles experiment

The suspension of CuO nanoparticles (Sigma-Aldrich, particle size <50 nm, mean 30 nm) was prepared according to Manusadžianas et al. (2012). Before fractionation, the cells were exposed to 100 mg/L nCuO suspensions for 30 min (three independent replicates). Preparation of algae, exposure conditions and Cu concentration measurements in cell fractions are described in detail in Manusadžianas et al. (2017).

### Statistical analysis

The obtained data were based on three individual experiments with three replicates each performed on different dates. To evaluate the variation in the measured activities of enzyme biomarkers in various cell fractions, the mean of the three experiments (*n* = 3) and 95% confidence intervals of normal distribution was calculated. Normality was checked by the Shapiro–Wilk test. To detect biomarker activity differences among the fractions, two data sets, distinctly for each biomarker, were analysed by one-way ANOVA, and the significance of the differences between mean values was calculated by Tukey’s *post hoc* test at *p* < 0.05. Before ANOVA, Levene’s test was applied for variance homogeneity. The analysis was carried out using the software PASW Statistics 18.0 (Predictive Analytics Software, IBM).

## Results and Discussion

**Figure 2:**
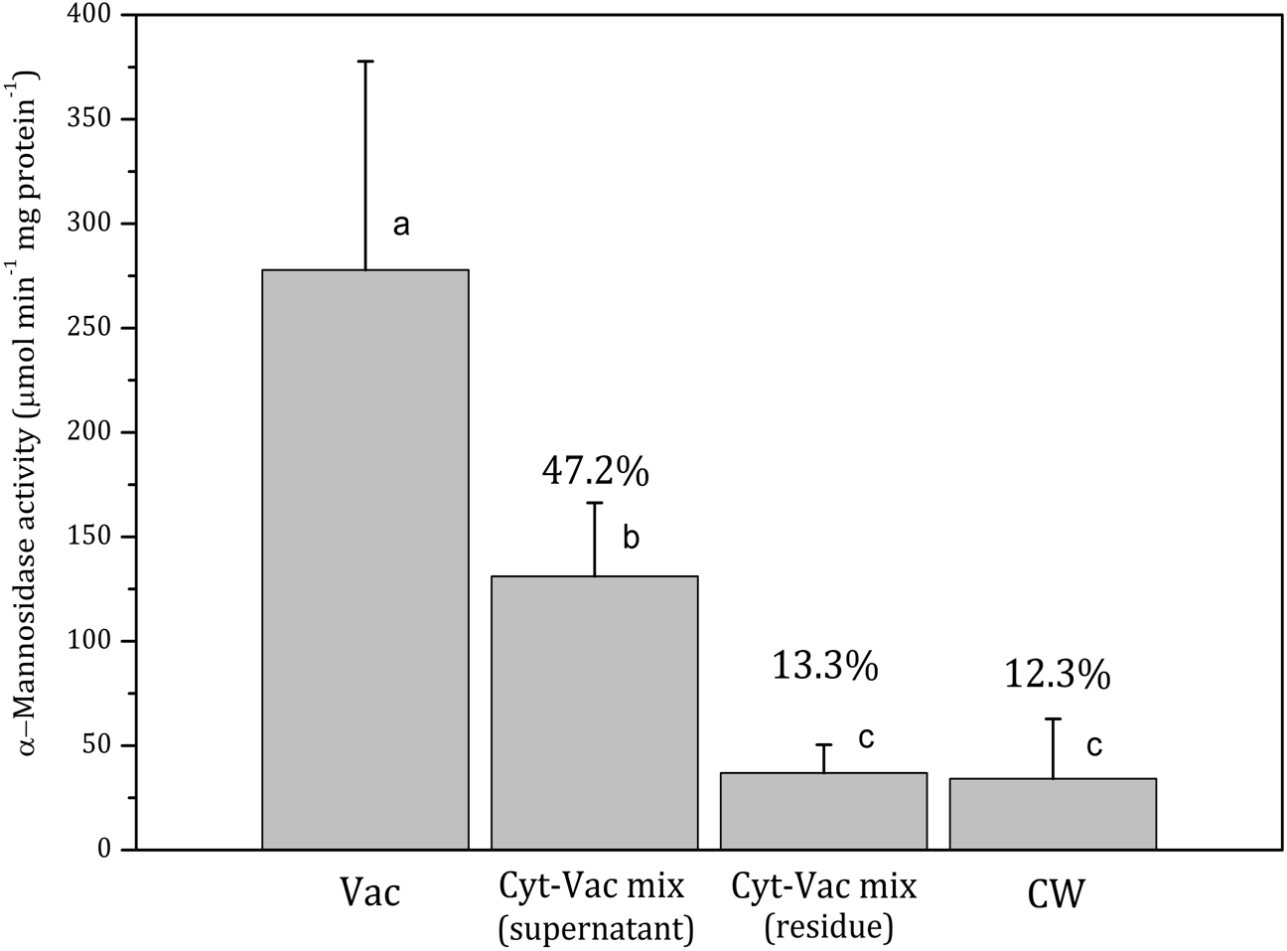
Activities of *α*-mannosidase in isolated fractions of *Nitellopsis obtusa* cells (mean ± 95% CI). Vac, vacuole; Cyt-Vac mix (supernatant), supernatant of centrifuged mixture of cytoplasm and vacuole; Cyt-Vac mix (residue), residue of centrifuged mixture of cytoplasm and vacuole; CW, cell wall. Percentages are presented in relation to *α*-mannosidase activity in vacuolar fraction (100%). Different letters indicate significant difference among the means (*p* < 0.05).

**Figure 3:**
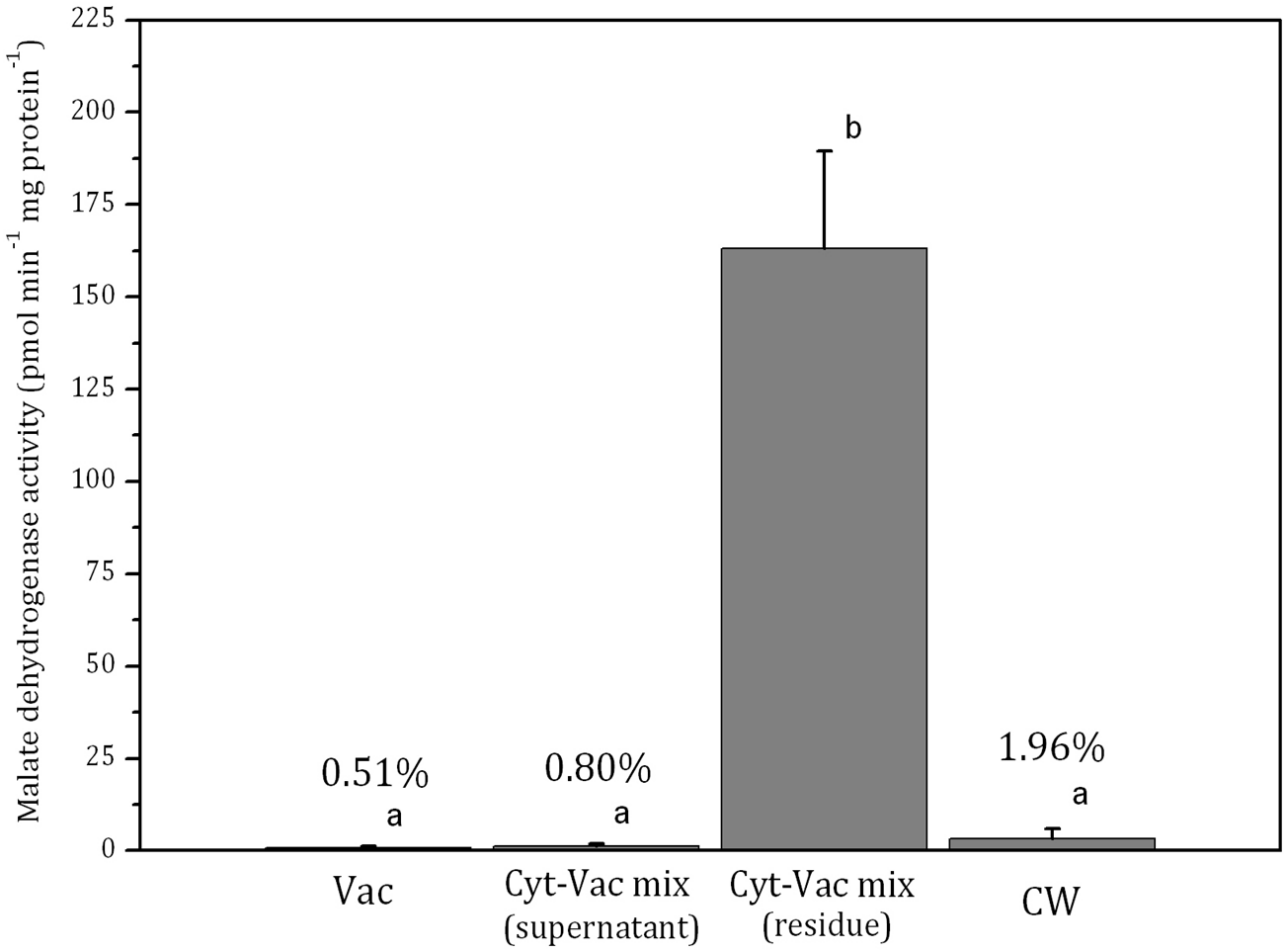
Activities of malate dehydrogenase in isolated fractions of *Nitellopsis obtusa* cells (mean ± 95% CI). Vac, vacuole; Cyt-Vac mix (supernatant), supernatant of centrifuged mixture of cytoplasm and vacuole; Cyt-Vac mix (residue), residue of centrifuged mixture of cytoplasm and vacuole; CW, cell wall. Percentages presented in relation to malate dehydrogenase activity in cytoplasmic fraction (100%). Different letters indicate significant difference among the means (*p* < 0.05).

Internodal cells of *Nitellopsis obtusa* were partitioned to obtain a vacuolar sap, the supernatant of the centrifuged cytoplasm-vacuole mix (without dripped vacuolar sap), the residue of centrifuged cytoplasm-vacuolar mix (without dripped vacuolar sap), total intracellular content and the cell wall. To confirm the purity of the isolated fractions and thus to attribute each of them to a particular compartment, the activities of two enzymes, *α*-mannosidase vacuolar biomarker (Fig. 2) and malate dehydrogenase cytoplasmic biomarker (Fig. 3) (Oikawa et al., 2011; Saunders & Gillespie, 1984), were determined for each fraction. The necessity to validate the compartment separation procedure came from our previous studies on CuO nanoparticles accumulation in the whole cell (Gylytė et al., 2015) and its main compartments (Manusadžianas et al., 2017). The effect mechanism of these nanoparticles to *N. obtusa* cells included a direct penetration of nCuO into the cell interior (Manusadžianas et al., 2012). However, the purity of obtained fractions and thus the procedure that has been used were not assessed quantitatively.

*α*-Mannosidase activity in vacuolar fraction comprised 278 ± 100 µmol min^−1^ mg protein^−1^ (mean ± 95% CI). The activity decreased more than half in the supernatant of centrifuged cytoplasm-vacuole mix (Cyt-Vac mix), leaving 47.2% of that of the vacuolar fraction (Fig. 2). The lowest *α*-mannosidase activities were measured in the residue of Cyt-Vac mix and cell wall fractions, respectively 13.3% and 12.3% concerning that of the vacuole, which shows a low degree of contamination of these fractions by vacuolar sap.

The activity of malate dehydrogenase in the residue of Cyt-Vac mix comprised 163 ± 26 pmol min^−1^ mg protein^−1^ (mean ± 95% CI) (Fig. 3). The activities in other fractions were negligible, i.e., comprising 0.51%, 0.80% and 1.96%, respectively in the vacuole, the supernatant of Cyt-Vac mix and the cell wall, to that measured in the residue of Cyt-Vac mix fraction (Fig. 3). The results show very low contamination of vacuole and cell wall fractions by the cytoplasm material. Concerning the supernatant of Cyt-Vac mix, it has to be mentioned that marginal activity of malate dehydrogenase measured in this fraction suggests very low or absence of cytoplasm material in that supernatant. The properties of the supernatant of the Cyt-Vac mix would largely depend on whether the chloroplasts remain intact or broken after cell squeezing. Photosynthetic pigments, i.e., chlorophylls a and b, and total carotenoids (Car), were analysed to determine whether Vac and Cyt-Vac mix (supernatant) fractions were contaminated with chloroplast debris. The results obtained showed that the fractions contained only negligible concentrations of some of the analysed pigments (in µg mL^−1^, mean ± sd, *n* = 3): 1.8 ± 0.1 (Chl a) and 6.2 ± 0.4 (Chl b) in Vac fraction, and 2.3 ± 0.1 (Chl b) in Cyt-Vac fraction. All this allowed assigning the residue of centrifuged Cyt-Vac mix to the cytoplasm compartment.

Decreased *α*-mannosidase activity in the supernatant of the cytoplasm-vacuole mix (Fig. 2) can be partially explained by the shift of pH value from 5.4 to 5.65 during the isolation procedure. It has been found that the maximum activity of *α*-mannosidase isolated from various plants is around pH 5, and the activity decreases sharply with the decrease in acidity (Hamayasu et al., 1997; Tejavath & Nadimpalli, 2014; Woo et al., 2004). The *α*-mannosidase activity in the residue of the Cyt-Vac mix (13.3%, Fig. 2) can be linked to contamination by vacuolar sap. However, we cannot strictly exclude the presence of *α*-mannosidase originating from ER (Van Der Wilden & Chrispeels, 1983) or other organelles, e.g., Golgi (De Marchis, Bellucci & Pompa, 2013). It should be mentioned that *α*-mannosidase activity has been measured in the ER of the seeds of *Phaseolus vulgaris* (Van Der Wilden & Chrispeels, 1983), but it has not been confirmed for mature plants. In our study, corresponding enzyme activities measured in the cell wall fraction revealed 12.3% contamination by vacuolar sap (Fig. 2) and 1.96% contamination by cytoplasm (Fig. 3). It seems that we overestimated the percent of *α*-mannosidase activity in the cell wall fraction compared to that of cytoplasm since the cell wall fraction was considered to include the nodes. The nodal complex consists of numerous small cells (Fig. 1C) that have their vacuoles. To check a possible influence of the vacuoles of the nodal cells, we explored *α*-mannosidase activity in the cell wall tubes with or without the nodes. Indeed, *α*-mannosidase activity measured in the cell wall tubes was approximately 1.7-fold lower than that in the cell walls including nodes, respectively, 27.1 ± 13.6 and 46.8 ± 22.2 µmol min^−1^ mg protein^−1^ (mean ± sd, *n* = 3).

By using similar compartment separation technique and enzymatic markers in the cells of *Chara australis*, Oikawa et al. (2011) have reported the isolation of high-purity vacuolar and cytoplasm content compartments, 99.9% and 93.1%, respectively. Our results with *N. obtusa* cells indicated 99.5% and 86.7% purity of vacuolar and cytoplasmic compartments as well as cell wall compartment contaminated slightly by cytoplasmic and vacuolar material. It has to be mentioned that the Japanese team has separated the internodal cell into two parts, the single vacuole, and the rest of the cell, including the cell wall. Besides, according to our results, the total intracellular content showed 8.50 ± 2.49 pmol min^−1^.mg protein^−1^ activity of malate dehydrogenase (mean ± sd, *n* = 3), i.e., comprising 5.2% in relation to malate dehydrogenase activity of that of cytoplasm. Thus, the volume of the vacuole of *N. obtusa* internodal cell can be accounted for 95% of the total cell volume. The data coincide with the radioactively labelled ion fluxes measurements in *N. obtusa* based** on which the volume of cytoplasm occupies up to 5% of that of the cell (MacRobbie & Dainty, 1958). For *Nitella*, it has been found that cytoplasm occupies at least 10% of the physical volume of the cell (Saltman & Christensen, 1961).

To summarise, after examining the fractionation procedure and evaluating the purity of isolated cell fractions by using enzymatic markers, we can identify the fractions as belonging to main cell compartments, i.e., the vacuolar sap (99.5% purity) represents high purity vacuole; the supernatant of Cyt-Vac mix (without dripped vacuolar sap) represents vacuole with lowered, due to changing acidity, *α*-mannosidase activity; the residue of centrifuged Cyt-Vac mix represents cytoplasm (86.7% purity); total intracellular content represents 5.2% of cytoplasm; and the cell wall (contaminated by 12.3 and 1.96% of vacuole and cytoplasm, respectively).

In our previous work (Manusadžianas et al., 2017), a similar cell fractionation technique has been applied to assess the location of accumulated CuO nanoparticles in the compartments of *N. obtusa* cells treated by 100 mg/L nCuO for 3 h. In the present study, we report data obtained after exposure of the cells for 0.5 h. The calculations in Table 1 have been done based on 10% cytoplasm volume of the whole cell volume (before re-estimation) and taking 5.2% of the cytoplasm volume based on the findings of the present study (after re-estimation). The data confirm that the internalisation of CuO nanoparticles occurs already after 0.5 h exposure to nCuO and does not significantly differ from that of 3 h irrespective of the compartment. Re-estimation showed a substantial shift in accumulation of CuO nanoparticles within the compartments. This is obvious with the cytoplasm where Cu concentration increased roughly as much as twice at both exposure times.

**Table 1:** Copper concentration in the compartments of *N. obtusa* cells treated by 100 mg/L nCuO for 30 minutes and 3 hours. Data represent mean standard deviation of three independent replicates. The data in 4th column (*) are from a previous study (Manusadžianas et al., 2017).

Compartment/Exposure time	30 min		3 h	
	before re-estimation	after re-estimation	before re-estimation*	after re-estimation
Cell wall (mg/g DW)	0.93 ± 0.57	0.93 ± 0.57	1.22 ± 0.23	1.22 ± 0.23
Vacuole (mg/L)	0.14 ± 0.09	0.13 ± 0.07	0.12 ± 0.04	0.18 ± 0.05
Cytoplasm (mg/L)	1.30 ± 0.37	2.39 ± 0.74	1.44 ± 0.55	2.98 ± 1.17

In general, isolation methods used in higher plant physiology involve multistep floatation gradient-, density gradient- or differential centrifugation. The technique discussed in our study allows separating internodal cell of *N. obtusa* into three compartments: vacuolar, cytoplasmic (with chloroplasts and other membranes) and cell wall. It has some advantage over other methods. In particular, the perfusion method (Sakano & Tazawa, 1984) or isolation of the vacuole from higher plant protoplasts (Robert et al., 2007; Skaliter et al., 2018) are superior methods for isolating a pure, single vacuole from a single mature cell; however, the first one is somewhat time-consuming, while another is also quite expensive. The procedure described in the present study can be conducted on turgorless cells within seconds.

## Conclusions

Isolating of charophyte cell main compartments, i.e., vacuole, cytoplasm and cell wall by using mechanical manipulation proved to be a reliable method for separating different fractions of *N. obtusa* cell. Application of *α*-mannosidase and malate dehydrogenase, vacuolar and cytoplasmic biomarkers, respectively, confirmed high purity of obtained vacuolar (99.5%) and cytoplasmic (86.7%) fractions. Cell wall fraction was contaminated slightly by vacuole and cytoplasm residues, respectively 12.3 and 1.96% of corresponding biomarker activities. The higher contamination by vacuolar component in the cell wall fraction could be caused by the vacuoles from the numerous small cells in the nodes. Estimation of the purity of mechanically separated cell fractions enabled reevaluating CuO related Cu concentrations in the compartments of charophyte cell.

##  Supplemental Information

10.7717/peerj.10930/supp-1 Supplemental Information 1 Biomarker activities and Cu concentrations in charophyte cell compartments Raw data of enzymatic biomarker activities and Cu measurements in isolated cell fractions.

## Additional Information and Declarations

### Data Availability

The following information was supplied regarding data availability: Raw data of enzymatic activities of a-mannosidase and malate-dehydrogenase in the fractions isolated from the cells of *Nitellopsis obtusa* are available as a Supplemental File.
